# PANoptosis-related molecular subtype and prognostic model associated with the immune microenvironment and individualized therapy in pancreatic cancer

**DOI:** 10.3389/fonc.2023.1217654

**Published:** 2023-07-14

**Authors:** Biao Zhang, Bingqian Huang, Xiaonan Zhang, Shuang Li, Jingyi Zhu, Xu Chen, Huiyi Song, Dong Shang

**Affiliations:** ^1^ Department of General Surgery, Clinical Laboratory of Integrative Medicine, The First Affiliated Hospital of Dalian Medical University, Dalian, China; ^2^ Institute (College) of Integrative Medicine, Dalian Medical University, Dalian, China

**Keywords:** pancreatic cancer, PANoptosis, pan-cancer analysis, molecular subtypes, prognostic model, tumor microenvironment, drug sensitivity

## Abstract

**Background:**

PANoptosis is an inflammatory type of programmed cell death regulated by PANopotosome. Mounting evidence has shown that PANoptosis could be involved in cancer pathogenesis and the tumor immune microenvironment. Nevertheless, there have been no studies on the mechanism of PANoptosis on pancreatic cancer (PC) pathogenesis.

**Methods:**

We downloaded the data on transcriptomic and clinical features of PC patients from the Cancer Genome Atlas (TCGA) and Gene Expression Omnibus databases. Additionally, the data on copy number variation (CNV), methylation and somatic mutations of genes in 33 types of cancers were obtained from TCGA. Next, we identified the PANoptosis-related molecular subtype using the consensus clustering analysis, and constructed and validated the PANoptosis-related prognostic model using LASSO and Cox regression analyses. Moreover, RT-qPCR was performed to determine the expression of genes involved in the model.

**Results:**

We obtained 66 PANoptosis-related genes (PANRGs) from published studies. Of these, 24 PC-specific prognosis-related genes were identified. Pan-cancer analysis revealed complex genetic changes, including CNV, methylation, and mutation in PANRGs were identified in various cancers. By consensus clustering analysis, PC patients were classified into two PANoptosis-related patterns: PANcluster A and B. In PANcluster A, the patient prognosis was significantly worse compared to PANcluster B. The CIBERSORT algorithm showed a significant increase in the infiltration of CD8^+^ T cells, monocytes, and naïve B cells, in patients in PANcluster B. Additionally, the infiltration of macrophages, activated mast cells, and dendritic cells were higher in patients in PANcluster A. Patients in PANcluster A were more sensitive to erlotinib, selumetinib and trametinib, whereas patients in PANcluster B were highly sensitive to irinotecan, oxaliplatin and sorafenib. Moreover, we constructed and validated the PANoptosis-related prognostic model to predict the patient’s survival. Finally, the GEPIA and Human Protein Atlas databases were analyzed, and RT-qPCR was performed. Compared to normal tissues, a significant increase in *CXCL10* and *ITGB6* (associated with the model) expression was observed in PC tissues.

**Conclusion:**

We first identified the PANoptosis-related molecular subtypes and established a PANoptosis-related prognostic model for predicting the survival of patients with PC. These results would aid in exploring the mechanisms of PANoptosis in PC pathogenesis.

## Introduction

Pancreatic cancer (PC) is one of the malignant tumors with the worst prognosis, with 95% of pathological types being pancreatic ductal adenocarcinoma (PDAC), which is characterized by rapid progression, high tendency to metastasis and spread, high resistance to various anti-cancer treatments, and an overall 5-year survival rate of only 11% (1–3). Due to the subtle nature of symptoms, only 20% of patients are diagnosed early. Most patients are diagnosed at an advanced stage when the symptoms are obvious. The survival rate of patients with advanced-stage PC is low since these patients are ineligible for radical surgery (4, 5). Additionally, the tumor microenvironment (TME) of patients with PC is complex; hence, the success rate of adjuvant therapy is low compared to surgery (6). PC has a prominent matrix of connective tissue hyperplasia, which restricts immune cell infiltration, anti-tumor responses, and adequate drug diffusion. Furthermore, the molecular characteristics of different PC subtypes are different. Epithelial markers are primarily expressed by classical PC subtypes. Moreover, basal-like subtypes are poorly differentiated and characterized by mesenchymal markers, such as laminin and basal keratin expression, stem cells, and epithelial-to-mesenchymal transition markers. Compared to the classical subtypes, the prognosis and response of patients with basal-like PC subtype to anticancer drugs are poor (7). Therefore, understanding the pathogenesis of PC and identifying its different molecular subtypes can help predict prognosis and design new therapeutic strategies.

Various studies have widely evaluated the involvement of inflammation in tumorigenesis. Studies showed systemic and chronic local inflammation could increase PC risk and the infiltration of inflammatory factors in the TME of patients with PC, thereby enhancing the growth and metastasis of cancer cells (8). PANoptosis is a newly discovered type of inflammatory programmed cell death regulated by PANopotosome. PANoptosis has key features of three types of cell death pathways, including apoptosis, pyroptosis, and necroptosis, but cannot be distinguished by a specific characteristic of any of these forms of cell death alone (9, 10). A study has shown the simultaneous activation of three types of cell death pathways in macrophages infected with the influenza virus. These pathways regulate each other, and if one pathway is blocked, the other pathway initiates a compensatory response (11). Several studies have shown the involvement of PANoptosis in tumorigenesis and tumor immune microenvironment (TIME) (12–14). ADAR1 and ZBP1 contain the Z-α domain, which is critically involved in innate immunity (15). ZBP1 triggers PANoptosis activation *via* the RIPK3 signaling pathway. Moreover, ADAR1 negatively regulates PANoptosis mediated by ZBP1; hence, attenuating ADAR1 activity could aid in inhibiting tumorigenesis (16, 17). The members of the caspase family are involved in cell death, inflammation, and innate immunity (18). Additionally, a study has shown that the proinflammatory caspases can convert the precursors of IL-1β and IL-18 into the secreted and bioactive forms, further recruiting more inflammatory cells and enhancing the inflammatory response to promote pyroptosis (19). Recent studies have shown that caspase-8 and caspase-6 were key proteins in the PANoptosis crosstalk signaling pathway (20, 21). In addition to mediating the intrinsic apoptosis, caspase-8 can also mediate pyroptosis by cleaving gasdermin family proteins. Furthermore, expression of enzymatically inactive caspase-8 can lead to embryonic death and inflammatory tissue destruction in mice by inducing necroptosis and pyroptosis. Caspase-6 was initially reported to mediate apoptosis. With the continuous exploration of programmed cell death, caspase-6 was found to mediate pyroptosis by regulating gasdermin D and necroptosis by regulating mixed lineage kinase domain-like. Molecular characteristics associated with PANoptosis could predict the survival and response of patients with gastric and colorectal cancers to immunotherapy (22, 23). However, no studies have shown the involvement of PANoptosis in patients with PC.

In this study, we identified PANoptosis-related genes (PANRGs) and analyzed the genetic alterations in PANRGs in pan-cancer. Subsequently, we constructed the PANoptosis-related molecular subtypes and prognostic model for patients with PC. Finally, we performed extensive bioinformatics analysis and experimental validation. Our results demonstrated an association between PANoptosis and the occurrence, the patient’s clinicopathological features, prognosis, biological behavior, TIME, and response to anticancer drugs in PC. These results would enhance our understanding of the mechanism of PANoptosis and design new strategies for treating patients with PC.

## Materials and methods

### Data acquisition and preprocessing

We obtained the transcriptomic and clinical data (containing 185 patients with PC) from the Cancer Genome Atlas (TCGA) database (https://portal.gdc.cancer.gov/). The data on copy number variation (CNV), methylation, and somatic mutations in 33 types of cancer were obtained from TCGA for exploring the genetic changes in PANRGs in pan-cancer. In addition, we retrieved the GSE62452 dataset [containing survival data of 69 PC samples, gene expression data of tumor tissues (TT) and adjacent tissues of 69 patients with PC], the GSE28735 dataset (consisting of data on survival data of 45 PC samples, gene expression data of TT and adjacent tissues of 45 patients with PC), the GSE85916 dataset (containing data on survival and gene expression in 80 patients with PC), and the GSE57495 dataset (containing gene expression and survival data of 63 patients with PC) from the Gene Expression Omnibus (GEO) database (https://www.ncbi.nlm.nih.gov/geo/). Further, we obtained data on the gene expression of normal pancreatic tissue from 167 healthy individuals from the Genotype-Tissue Expression Project (GTEx) *via* the UCSC Xena platform (https://xenabrowser.net/datapages/). We eliminated the batch effects in different datasets using the “sva” R package (24). PANRGs were obtained from a previously published study (23). We excluded patients with survival duration < 30 days. Finally, we screened PANRGs-related to patient prognosis using univariate Cox regression analysis for subsequent studies.

### Clustering analysis

Based on PANRG expression, all patients from TCGA, GSE28735, GSE62452, GSE57495, and GSE85916 datasets were pooled and clustered using the “ConsensusClusterPlus” package to determine the PANoptosis-related molecular subtypes of PC (25). We determined the optimal cluster number using the consensus matrix and the cumulative distribution function (CDF) curve. Further, we reduced dimensionality and determined the reliability of clustering using the principal component analysis (PCA) (26). The survival duration of patients in different subtypes was compared using the Kaplan-Meier (KM) survival curves. We screened differentially expressed genes (DEGs) in patients in different subtypes using the “limma” package to determine the PANoptosis-associated DEGs (PANDEGs). These PANDEGs were screened based on the following criteria: “|log2FC| > 1” and “adjusted *P* < 0.05”. Finally, we investigated functions and processes enriched by PANDEGs using Gene Ontology (GO) enrichment analysis.

### Constructing and validating PANoptosis-related prognostic model

We classified patients from TCGA randomly in a 5:5 ratio into the training and internal verification sets using the “caret” R package. Patients from GEO were used as the external verification set. First, we screened for PANDEGs related to prognosis using univariate Cox regression analysis. Next, the least absolute shrinkage and selection operator (LASSO) regression analysis was used to eliminate overfitting between genes (27). Finally, multivariate Cox regression analysis was performed to construct the PANoptosis-related prognostic model. The formula for calculating the PANscore was as follows: $\text{PANscore }=\;\sum_{i}\text{Coefficient }\left({\text{gene}}_{\text{i}}\right)*\text{mRNA Expression }\left({\text{ gene}}_{\text{i}}\right)$ . In the training set, all patients were classified using the median PANscore as a threshold value into the high-PANscore group (HPSG) and low-PANscore groups (LPSG). The KM survival curve was used for comparing the survival duration of patients in both groups. The PANoptosis-related prognostic model was evaluated using the time-dependent Receiver Operating Characteristic (ROC) curve and the area under the ROC curve (AUC).

### Correlation between clinicopathological features and independent prognostic analysis

The data on clinicopathological features were available for patients with PC from TCGA. Hence, we combined the clinicopathologic features and PANscore of these patients from TCGA. Next, we compared the PANscore of patients in different clinicopathological groups using the Wilcoxon signed-rank test. Finally, univariable and multivariable Cox regression analyses were used to determine if PANscore could independently predict the patient’s prognosis.

### Gene set variation analysis, immune cell infiltration, and drug sensitivity

GSVA is used to study the biological behavior of patients by calculating gene set enrichment scores in all patients (28). We determined the biological behavior of patients in different molecular subtypes or PANscore groups using GSVA based on the “c2.cp.kegg.v7.5.1.symbols.gmt” gene set. The characteristics of the TIME in patients in different molecular subtypes or PANscore groups were evaluated *via* the Cell-type Identification by Estimating Relative Subsets of RNA Transcripts (CIBERSORT) algorithm by determining the infiltration of 22 immune cell subtypes in all patients (29). Samples with *P* < 0.05 indicated that the assessment of infiltrating immune cells was accurate, and these samples were used for subsequent studies. Further, we used the “OncoPredict” R package to predict the drug response *in vivo* or in cancer patients based on data screened using cell lines (30). Finally, we utilized the “OncoPredict” R package to evaluate the differences in the sensitivity of patients in different molecular subtypes or PANscore groups to drugs.

### Expression, prognostic value, and distribution of genes in the model

GEPIA (http://gepia.cancer-pku.cn/) is a web-based portal for determining gene expression, correlation, and prognosis of patients from TCGA and GTEx (31). We used the GEPIA database to determine the difference in the expression of the PANoptosis-related prognostic model genes in pancreatic TT and normal tissues (NT). The Human Protein Atlas (HPA) database (https://www.proteinatlas.org/) is a web-based platform. It is used to study the expression patterns of proteins in cells and tissues (32). We used the HPA database to determine the difference in protein expression and subcellular localization of PANoptosis-related prognostic model genes in pancreatic TT and NT. The Tumor Immune Single-cell Hub (TISCH) database (http://tisch.comp-genomics.org) is used to study the TME at the single-cell level (33). TISCH was used to study the expression of PANoptosis-related prognostic model genes in cell types of TME of patients with PC.

### Cell lines and organoids

HPDE6-C7, pancreatic ductal epithelium cells of human origin, were purchased from the American Type Culture Collection (Manassas, VA, USA). PC cell lines, including PANC-1, CF-PAC1, BxPC-3, and MIAPaCa-2 of human origin, were obtained from Procell Life Science & Technology Co., Ltd and the KeyGEN BioTECH (Jiangsu province, China). BxPC-3 and CF-PAC1 cells were cultured in RPMI-1640 and Iscove’s modified Dulbecco medium, respectively, supplemented with 10% fetal bovine serum (FBS, Gibco, Carlsbad, CA, USA). HPDE6-C7, MIAPaCa-2, and Panc-1 cells were cultured in Dulbecco’s modified Eagle’s medium (DMEM) (Gibco) supplemented with 10% FBS (Gibco). All cells were cultured at 37 °C, 95% air, and 5% CO_2_.

Tissues for establishing PC organoids and biological analyses were obtained from patients from the first affiliated hospital of Dalian Medical University. All procedures involving human participants were approved by the institutional ethics committee. All patients or their legal guardians provided informed consent to participate in the study. First, we harvested pancreatic tissues by surgical resection and cut them into nearly 1 cm^2^ fragments. Next, the tissues were washed until the supernatant appeared clear using the cold chelation buffer. Further, the fragments were enzymatically digested using I.5 mg/mL of collagenase (Gibco) and 20 µg/mL hyaluronidase (Sigma) in 10 mL advanced DMEM-F12 (Gibco) + antibiotics (Primocin, Invivogen, San Diego, CA) for 1 h at 37°C on a shaker. Cells were washed twice with advanced DMEM-F12, seeded on Matrigel, and cultured in a medium containing HEPES, Penicillin, Glutamax, Streptomycin, B27, EGF, TGFβ-inhibitor, R-spondin1, Wnt, FGF10, Noggin, n-Acetylcysteine, Gastrin, and RHOK-inhibitor.

### Real-time quantitative PCR

We extracted total cellular RNA from cell lines and organoids, synthesized cDNA *via* reverse transcription, and performed RT-qPCR to determine gene expression using a qPCR Kit (Accurate Biotechnology). The reagents used for the experiments came from our laboratory. We used *GAPDH* as the control standard. Finally, gene expression was analyzed and quantified using the ΔΔCt method. All primers corresponded to human genes and were obtained from GenePharma (Suzhou, China). The primer sequences were as follows: CXCL10: Forward primer: 5’-AGGGTGAGAAGAGATGTCTGAATCC-3’, Reverse primer: 5’-AGACCTTTCCTTGCTAACTGCTTTC-3’; ITGB6: Forward primer: 5’-TGTATCTGCCACTTGTCTCCCTATG-3’, Reverse primer: 5’-ACAGTCACAGTCGCCGTTACC-3’.

### Statistical analysis

The analysis and visualization of data were performed using the R (Ver 4.1.2) and GraphPad Prism 9 software. First, the differences in data in two groups with normal distribution were compared using the *t*-test. Next, the difference in data in the two groups not obeying normal distribution was compared using the Wilcoxon rank-sum test. Next, we determined the correlation using Spearman or Pearson analysis. Finally, the survival duration of patients in different groups was compared using the KM survival analysis. *P* < 0.05 was considered statistically significant.

## Results

### Screening PANRGs and pan-cancer analysis

**Figure 1:**
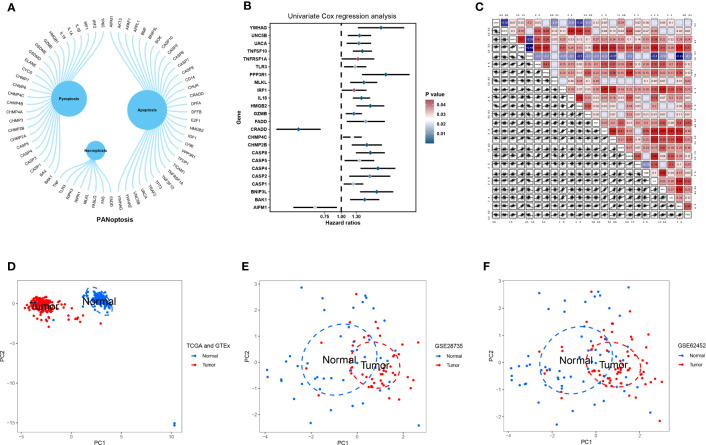
Acquisition and analysis of PANRGs. **(A)** A total of 66 PANRGs, including eight necroptosis-related genes, 26 pyroptosis-related genes, and 32 apoptosis-related genes, were identified. **(B)** Univariate Cox regression analysis showed that 24 PANRGs were associated with the prognosis of patients with PC. **(C)** Correlations between 24 PANRGs. PCA showed that PANRGs could distinguish between TT and NT of PC patients from TCGA and GTEx **(D)**, GSE62452 **(E)**, and GSE28735 **(F)** datasets.

We obtained 66 PANRGs from a published study, of which eight were necroptosis-related genes, 26 were pyroptosis-related genes, and 32 were apoptosis-related genes (**Figure 1A**). Univariate Cox regression analysis revealed 24 PANRGs significantly associated with patient prognosis, of which most genes were risk factors. *CRADD* and *AIFM1* were protective factors for the patient’s prognosis (**Figure 1B**). The results showed that *CRADD* was positively correlated with *AIFM1*, and *CRADD* was negatively correlated with the other 22 PANRGs (**Figure 1C**). PCA revealed that PANRGs could significantly distinguish between pancreatic TT and NT of patients from TCGA and GTEx (**Figure 1D**), GSE62452 (**Figure 1E**), and GSE28735 (**Figure 1F**) datasets. These results indicate that PANRGs could be related to PC pathogenesis.

**Figure 2:**
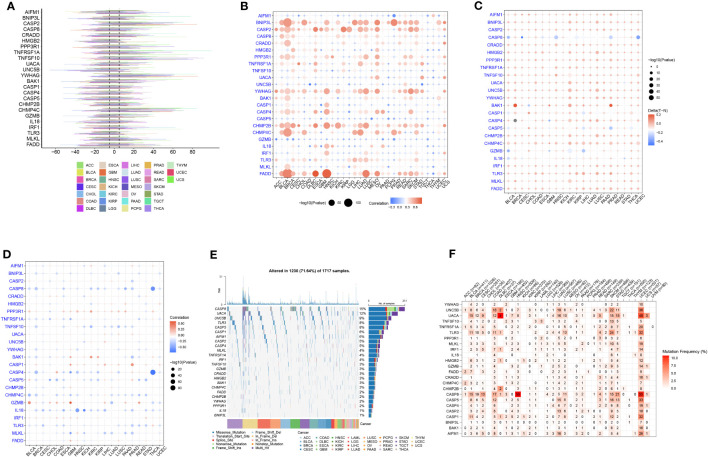
Pan-cancer analysis of PANRGs. **(A)** The frequency of CNV in PANRGs in pan-cancer. **(B)** The correlation between CNV and PANRG expression. **(C)** The difference in PANRG methylation in pancreatic TT and NT. **(D)** The correlation between methylation and PANRG expression. **(E)** The mutational landscape of PANRGs. **(F)** The frequency of mutations in PANRGs in 33 cancers.

Next, we analyzed the CNV, methylation, and mutational changes in PANRGs in pan-cancer. The results revealed that CNV in PANRGs was commonly found in 33 types of cancers. The frequency of CNV ranged from 5%–60%. *CASP2, TNFSF10, YWHAG*, and *CHMP4C* harbored CNV gains, and *HMGB2* and *TLR3* harbored CNV deletion (**Figure 2A**). Next, we explored the correlation between CNV and gene expression to study the regulatory effect of CNV on gene expression. The results showed that CNV in most PANRGs was significantly positively correlated with gene expression. However, CNV in *CASP5* and *GZMB* was negatively correlated with gene expression (**Figure 2B**). In addition to CNV, methylation affects gene expression and is associated with cancer development (34). The results revealed hypermethylation in most PANRGs in pancreatic TT compared to NT, except *CASP8* (**Figure 2C**). Furthermore, a complex correlation was observed between PANRG methylation and expression. The results showed a negative correlation between *BNIP3L, CASP8, CASP4, CHMP4C*, and *IL18* methylation and expression and a positive correlation between *PPP3R1* and *CASP1* methylation and expression (**Figure 2D**). Finally, we analyzed the mutational landscape of PANRGs in pan-cancer, and the results revealed mutations in all PANRGs, of which the mutation frequency was highest in *CASP8* (15%), *UACA* (12%), and *UNC5B* (9%, **Figure 2E**). A significantly high mutation frequency in PANRGs was observed in uterine corpus endometrial carcinoma, skin cutaneous melanoma, and stomach adenocarcinoma among these 33 types of cancers (**Figure 2F**).

### Identification and analysis of PANoptosis-related molecular subtypes

**Figure 3:**
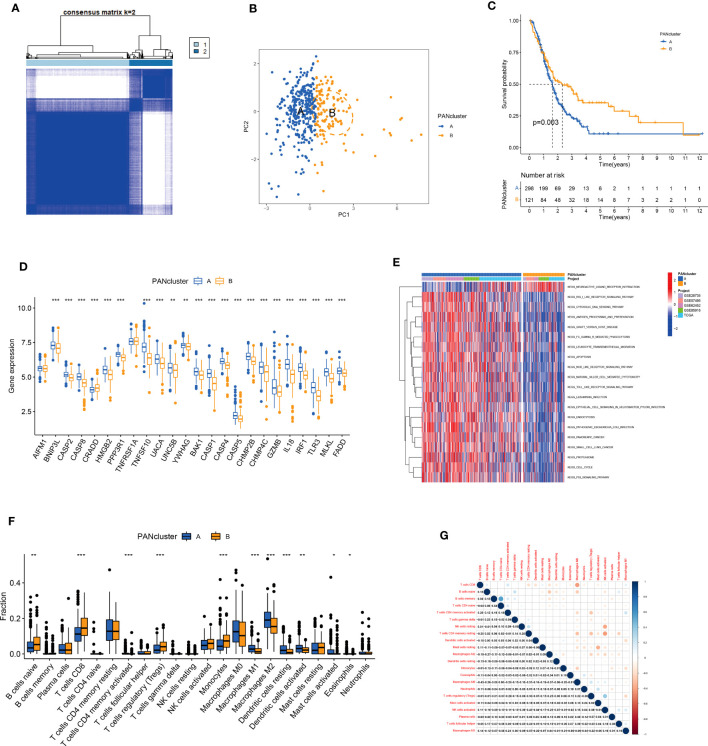
Identification and analysis of PANoptosis-related molecular subtypes. **(A)** Heatmap shows consensus matrix. **(B)** PCA could significantly distinguish between PANcluster **(A–C)** KM survival curve of patients in PANcluster **(A, B, D)** Difference in PANRG expression in PANcluster **(A, B, E)** GSVA. **(F)** The infiltration of 22 immune cell subtypes in patients in PANcluster **(A, B, G)** Correlations between 22 immune cell subtypes. (*p<0.05;**p<0.01;***p<0.001).

We performed the consensus clustering analysis on PANRG expression. The CDF curve and the changed area under the curve suggested that the optimal clustering number was two (**Figures S1**, **S2**). All patients with PC were classified into PANcluster A and PANcluster B (**Figure 3A**). PCA could significantly distinguish patients with PC in these two molecular subtypes, thereby indicating the reliability of the clustering (**Figure 3B**). The prognosis of patients in PANcluster A was significantly worse compared to PANcluster B (**Figure 3C**). Next, we determined PANRG expression in patients in both two subtypes. The results demonstrated a significant increase in the expression of most PANRGs and a significant decrease in *CRADD* expression in patients in PANcluster A (**Figure 3D**). Further, we performed GSVA to determine the functions and pathways enriched in patients in both molecular subtypes. The results demonstrated significant enrichment of the cytosolic DNA sensing, NOD-like receptor, and Toll-like receptor (TLR) signaling pathways, the processing and presentation of antigens, leukocyte transendothelial migration, apoptosis, and cytotoxicity mediated by natural killer (NK) cells in patients in PANcluster A (**Figure 3E**). Additionally, we employed the CIBERSORT algorithm to determine immune cells infiltrating the TME of patients in both subtypes. The results demonstrated a significant increase in the infiltration of naive B cells, CD8^+^ T cells, regulatory T cells, and monocytes in the TME of patients in PANcluster B. Moreover, a significant increase in the infiltration of M0, M1, and M2 macrophages, resting and activated dendritic cells (DCs), activated mast cells, and eosinophils was observed in patients in PANcluster A (**Figure 3F**). Further, the correlation analysis revealed a complex correlation between different immune cells. The results demonstrated that CD8^+^ T cells were positively correlated with naive B cells, activated memory CD4^+^ T cells, and M1 macrophages. However, a negative correlation exists between CD8^+^ T cells and resting memory CD4^+^ T cells, activated DCs, and M2 and M0 macrophages (**Figure 3G**).

**Figure 4:**
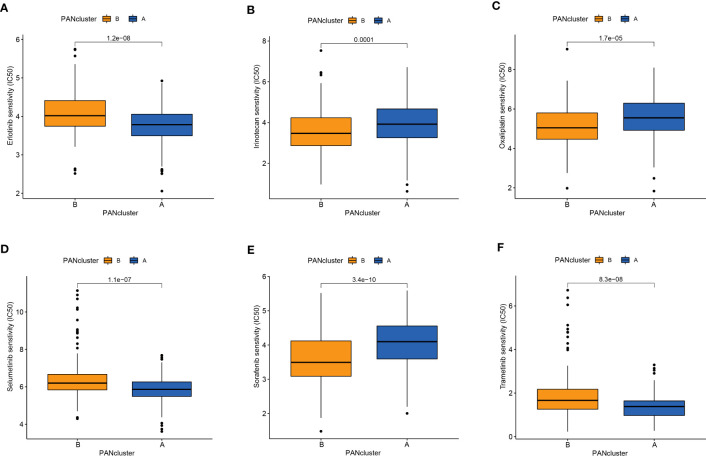
Drug sensitivity in patients in different PANoptosis-related molecular subtypes. Patients within PANcluster A were more sensitive to erlotinib **(A)**, selumetinib **(D)**, and trametinib **(F)**. Patients in PANcluster B were more sensitive to irinotecan **(B)**, oxaliplatin **(C)**, and sorafenib **(E)**.

Drug-assisted therapy, especially chemotherapy, is widely used for treating patients with PC. However, the responses of different patients with PC to chemotherapy are different. Therefore, screening patients based on their sensitivity to different drugs could aid clinical decision-making. The results revealed differences in the responses of patients in both molecular subtypes to chemotherapy. The patients in PANcluster A were highly sensitive to erlotinib, selumetinib, and trametinib (**Figures 4A, D, F**). Whereas patients in PANcluster B were highly sensitive to irinotecan, oxaliplatin, and sorafenib (**Figures 4B, C, E**).

### Screening for DEGs in patients in PANoptosis-related molecular subtypes

**Figure 5:**
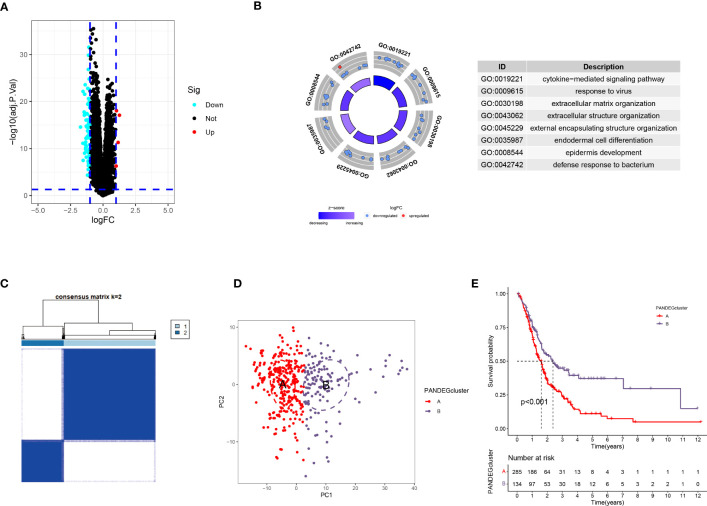
Differential expression analysis in patients in PANoptosis-related molecular subtypes. **(A)** A total of 73 PANDEGs were identified. **(B)** GO enrichment analysis of PANDEGs. **(C)** Heatmap shows consensus matrix. **(D)** PCA could significantly distinguish patients in PANDEGcluster **(A, B, E)** Survival curve of patients in PANDEGcluster **(A, B)**.

We screened for DEGs in patients to determine the differences between the different PANoptosis-related molecular subtypes. First, we identified 73 PANDEGs, of which 69 PANDEGs were significantly upregulated in PANcluster A, and four PANDEGs were upregulated in PANcluster B (**Figure 5A**). Next, we performed a GO enrichment analysis on these 73 PANDEGs to determine the functions and processes enriched by these PANDEGs. The results demonstrated significant enrichment of these 73 PANDEGs in the cytokine-mediated signaling pathway, the organization of extracellular matrix (ECM) and external encapsulating structures, response to the virus, and endodermal cell differentiation (**Figure 5B**). To study the effect of PANDEGs on the patient’s prognosis, we performed a consensus clustering analysis based on PANDEG expression. All patients with PC were divided into PANDEGcluster A and PANDEGcluster B (**Figure 5C**). PCA could distinguish patients with PC in PANDEGcluster A and PANDEGcluster B, thereby indicating the reliability of the clustering (**Figure 5D**). In addition, the prognosis of patients in PANDEGcluster B was significantly better compared to PANDEGcluster A (**Figure 5E**).

### Constructing and validating PANoptosis-related prognostic model

**Figure 6:**
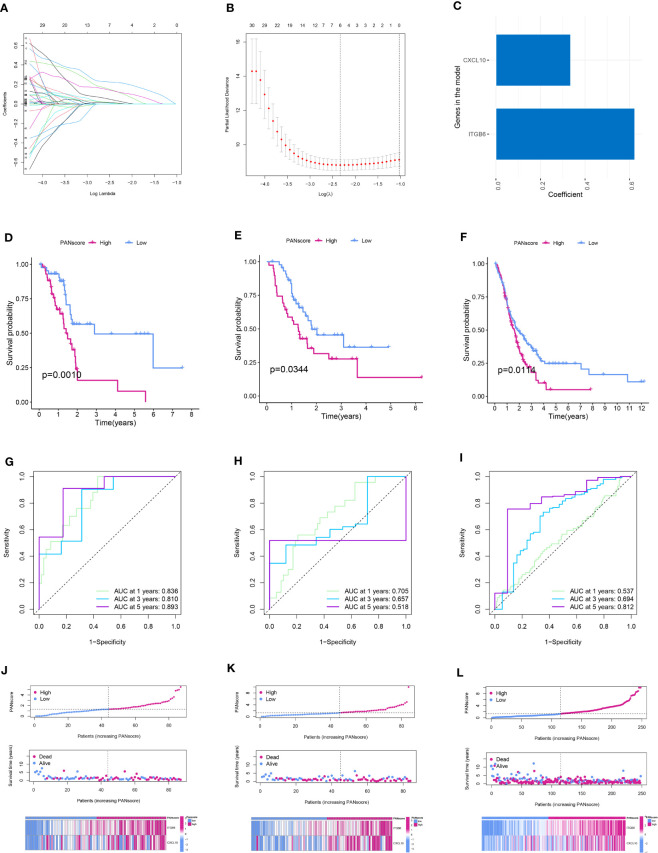
Establishment and validation of the PANoptosis-related prognostic model. **(A)** The coefficient path diagram of LASSO regression analysis. **(B)** The cross-validation curve of LASSO regression analysis. **(C)** Coefficient of the model genes *CXCL10* and *ITGB6*. Survival curve of patients in the low and high PANscore group in the training **(D)**, internal validation **(E)**, and external validation **(F)** sets. ROC curve of the training **(G)**, internal validation **(H)**, and external validation sets **(I)**. PANscore curve, the scatter plot of the distribution of survival status, and the heatmap of model gene expression in patients in the training **(J)**, internal validation **(K)**, and external validation **(L)** sets.

We performed LASSO and Cox regression analyses to construct a PANoptosis-related prognostic model based on PANDEGs and calculated PANscores for all patients with PC. Further, 64 genes related to patient prognosis were identified using univariate Cox regression analysis (**Figure S3**). Next, the overfitting between 64 genes was eliminated using LASSO regression analysis (**Figures 6A, B**). Finally, we employed multivariate COX regression analysis to construct a PANoptosis-related prognostic model: PANscore = (*CXCL10* * 0.335141107156712) + (*ITGB6* * 0.623064631293372) (**Figure 6C**). The survival duration of patients in LPSG was significantly longer compared to HPSG (**Figure 6D**). Further, we validated the reliability of the model in the internal and external validation sets (**Figures 6E, F**). The AUC values of 1, 3, and 5-year survival rates of patients were 0.836, 0.810, and 0.893, respectively, in the training set (**Figure 6G**), 0.705, 0.657, 0.518, respectively, in the internal validation set (**Figure 6H**), and 0.537, 0.694, and 0.812, respectively, in the external validation set (**Figure 6I**). These results indicated that the ability of our model in predicting prognosis was good. On the other hand, the mortality rate of patients in HPSG was high. In addition, the mortality rate of patients expressing high *CXCL10* and *ITGB6* levels was also high (**Figures 6J–L**).

### Correlation of clinicopathological characteristics and independent prognostic analysis

**Figure 7:**
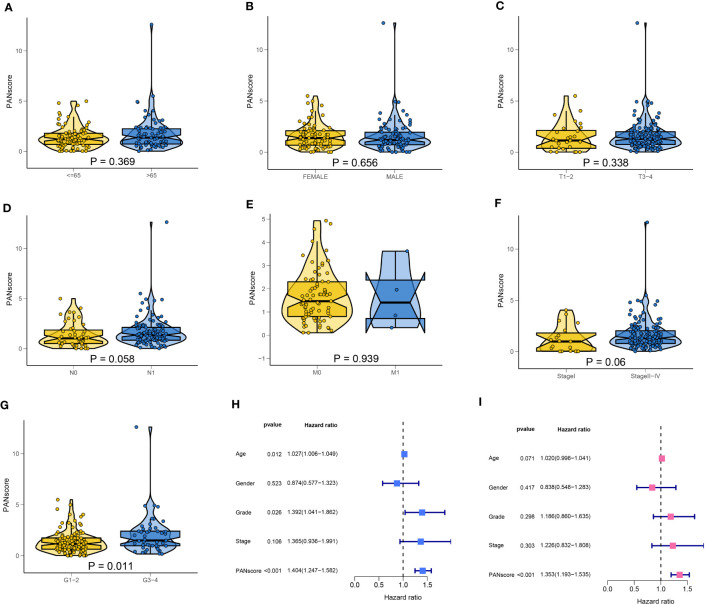
Correlation of clinicopathological characteristics and independent prognostic analysis. The PANscores of patients with different ages **(A)**, sex **(B)**, T stage **(C)**, N stage **(D)**, M stage **(E)**, TNM stage **(F)**, and grade **(G)**. **(H)** Univariate Cox regression analysis showed that age, pathological grade, and PANscore were associated with the prognosis of patients with PC. **(I)** Multivariate Cox regression analysis suggested that PANscore was an independent risk factor.

Next, we explored the correlation between PANscore and clinicopathological characteristics. The results revealed that PANscore was not significantly correlated with the patient’s age, sex, and T and M stages (**Figures 7A–C, E**). Patients with N1 stage PC had higher PANscore compared to patients with N0 stage PC, patients with Stage II-IV PC had higher PANscores compared to patients with Stage I PC, and the differences were close to statistical significance (**Figures 7D, F**). Additionally, a significant increase in PANscore of patients with Grade 3-4 PC was observed compared to patients with Grade 1-2 (*P* = 0.011, **Figure 7G**). Finally, we determined if PANscore could be a prognostic factor independent of clinicopathological features. Univariate Cox regression analysis demonstrated an association between age, pathological grade, PANscores, and patient’s prognosis (**Figure 7H**). Multivariate Cox regression analysis demonstrated that PANscore was an independent risk factor (**Figure 7I**).

### GSVA, immune cell infiltration, and drug sensitivity

**Figure 8:**
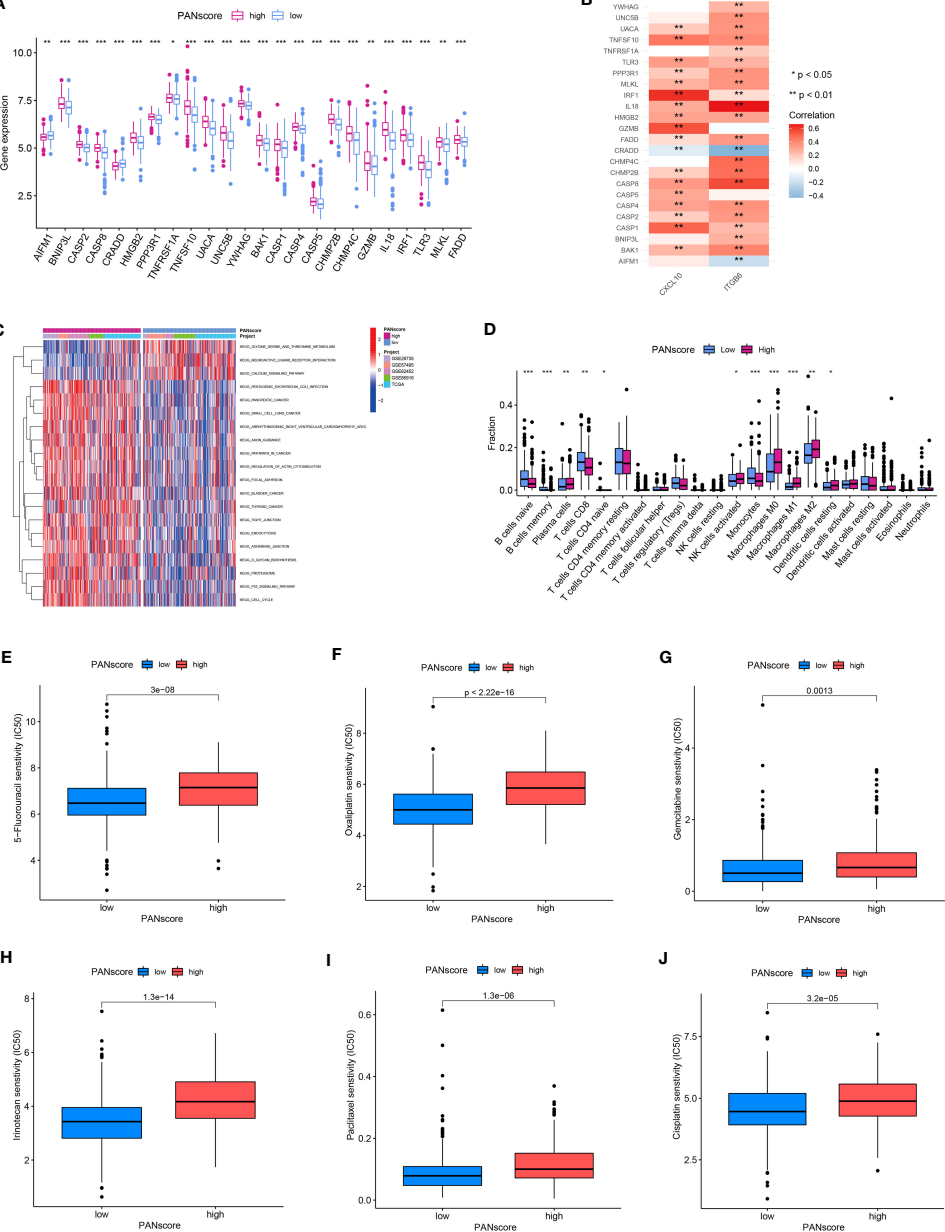
GSVA, immune cell infiltration, and drug sensitivity. **(A)** Differences in PANRG expression in patients in the HPSG and LPSG. **(B)** Correlations between PANRGs and model genes. **(C)** GSVA. **(D)** The infiltration of 22 immune cell subtypes in patients in both PANscore groups. Patients in LPSG were highly sensitive to 5-fluorouracil **(E)**, oxaliplatin **(F)**, gemcitabine **(G)**, irinotecan **(H)**, paclitaxel **(I)**, and cisplatin **(J)**. (*p<0.05;**p<0.01;***p<0.001).

Differential expression analysis showed a significant increase in most PANRG expression, and a significant decrease in *AIFM1* and *CRADD* expression was observed in patients in the HPSG (**Figure 8A**). We have demonstrated that *AIFM1* and *CRADD* were protective factors for patient prognosis. Therefore, we investigated the correlation between the model genes and PANRGs. The results revealed that *CXCL10* and *ITGB6* were positively correlated with most PANRGs and a significant negative correlation between *CXCL10* and CRADD. Moreover, *ITGB6* was significantly negatively correlated with *AIFM1* and *CRADD* (**Figure 8B**).

GSVA was performed to determine the difference in the biological processes enriched in patients in both PANscore groups. The results showed that pathogenic Escherichia coli infection, PC, small cell lung cancer, the regulation of actin cytoskeleton, focal adhesion, and the p53 signaling pathway were significantly enriched in patients in the HPSG. On the contrary, the serine, glycine, and threonine metabolism, neuroactive ligand-receptor infection, and the calcium signaling pathway were significantly enriched in patients in the LPSG (**Figure 8C**). Next, we determined immune cell infiltration in patients in both PANscore groups. The results demonstrated high infiltration of naïve and memory B cells, CD8^+^ T cells, and monocytes in patients in the LPSG. On the contrary, high infiltration of plasma cells, M0, M1, and M2 macrophages, and resting DCs were observed in the patients in HPSG (**Figure 8D**). Finally, we evaluated the differences in responses of patients with PC in different PANscore groups to drugs to determine the effect of PANscore on PC drugs sensitivity. The results showed that patients in the LPSG were highly sensitive to 5-fluorouracil, oxaliplatin, gemcitabine, irinotecan, paclitaxel, and cisplatin. These results would aid in designing personalized therapy for patients with PC (**Figures 8E–J**).

### Expression, distribution, and prognostic significance of model genes

**Figure 9:**
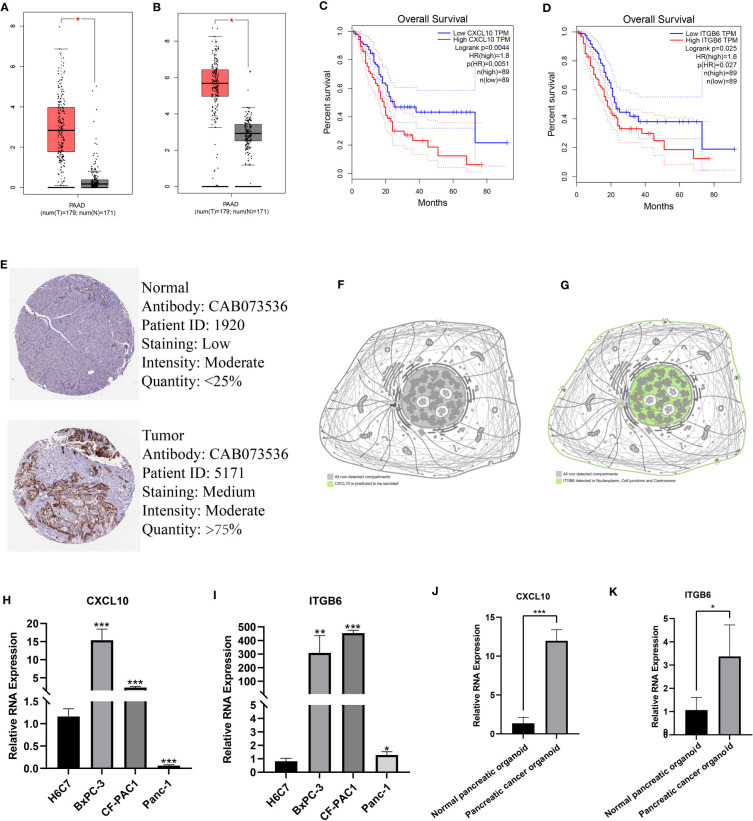
Expression, distribution, and prognostic significance of model genes. A significant increase in *CXCL10* **(A)** and *ITGB6* **(B)** expression in pancreatic TT compared to NT. The prognosis of patients expressing high *CXCL10* **(C)** and *ITGB6* **(D)** was significantly poor. **(E)** IHC images showed a significant increase in *ITGB6* expression in pancreatic TT compared to NT. **(F)** *CXCL10* was predicted to be secreted out of the cell. **(G)** *ITGB6* was localized in the nucleoplasm, cell junctions, and centrosome. RT-qPCR showed a significant increase in *CXCL10* **(H)** and *ITGB6* **(I)** expression in PC cells compared to normal pancreatic cells. A significant increase in *CXCL10* **(J)** and *ITGB6* **(K)** expression in PC organoids was found compared to organoids constructed using normal pancreatic tissues. (*p<0.05;**p<0.01;***p<0.001).

**Figure 10:**
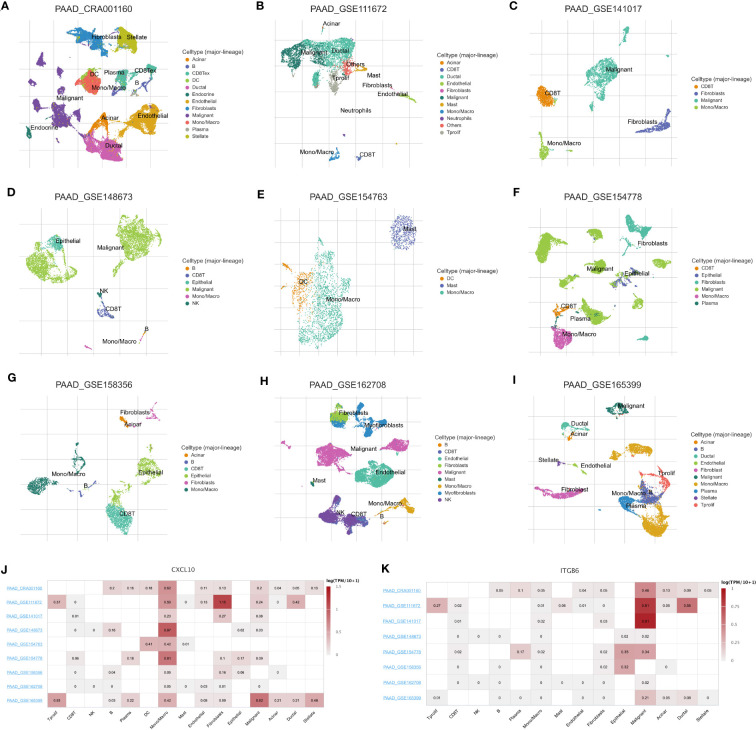
Single-cell analysis of PC. Annotation of all cell subsets from different datasets: CRA001160 **(A)**, GSE111672 **(B)**, GSE141017 **(C)**, GSE148673 **(D)**, GSE154763 **(E)**, GSE154778 **(F)**, GSE158356 **(G)**, GSE162708 **(H)** and GSE165399 **(I)**. **(J)** *CXCL10* was primarily expressed by monocytes/macrophages, cancer-associated fibroblasts, and cancer cells in the TME of patients with PC. **(K)** *ITGB6* was primarily expressed by cancer cells in the TME of patients with PC.

Finally, we determined the expression, distribution, and significance of *CXCL10* and *ITGB6* in predicting the patient’s prognosis. First, the GEPIA database was analyzed, and the results revealed a significant increase in *CXCL10* and *ITGB6* expression in pancreatic TT compared to NT (**Figures 9A, B**). Moreover, the prognosis of patients expressing high *CXCL10* and *ITGB6* levels was significantly poor (**Figures 9C, D**). We obtained immunohistochemistry (IHC) data from the HPA database. The results demonstrated a significant increase in *ITGB6* expression in pancreatic TT compared to NT (**Figure 9E**). Next, we investigated the localization of *CXCL10 and ITGB6* in subcellular structures, and found that *CXCL10* was predicted to be secreted (**Figure 9F**), whereas *ITGB6* was localized in the nucleoplasm, cell junctions, and centrosome (**Figure 9G**). Additionally, RT-qPCR results demonstrated a significant increase in *CXCL10* and *ITGB6* expression in PC cells compared to normal pancreatic cells (**Figures 9H, I**). Moreover, a significant increase in *CXCL10* and *ITGB6* expression in the PC organoids compared to the organoids was generated using normal pancreatic cells (**Figures 9J, K**). The TISCH database was used for single-cell analysis of nine single-cell datasets on PC from different sources: CRA001160 (**Figure 10A**), GSE111672 (**Figure 10B**), GSE141017 (**Figure 10C**), GSE148673 (**Figure 10D**), GSE154763 (**Figure 10E**), GSE154778 (**Figure 10F**), GSE158356 (**Figure 10G**), GSE162708 (**Figure 10H**) and GSE165399 (**Figure 10I**). *CXCL10* was mainly expressed by macrophages/monocytes, cancer-associated fibroblasts, and malignant cells (**Figure 10J**), and ITGB6 was primarily expressed by malignant cells in TME of patients with PC (**Figure 10K**).

## Discussion

PC is a common malignancy of the digestive system. In recent years, the burden of PC has increased globally, thereby posing a huge threat to human life, health, and the economy (35). Despite the efforts made in the past to diagnose and treat patients with PC, the prognosis of patients has not significantly improved. Therefore, an enhanced understanding of the pathogenesis of PC is required for screening novel approaches and treating patients with PC. PANoptosis is recently discovered as a novel inflammatory programmed cell death mechanism. It combines and regulates cell death pathways, such as apoptosis, pyroptosis, and necroptosis, by forming a PANoptosome as part of the innate immune responses of the host (36, 37). Recent studies have shown the significant involvement of PANoptosis in both tumorigenesis and anti-tumor therapy by regulating key regulatory elements of PANoptosis to inhibit tumorigenesis (37, 38). PANoptosomes can influence inflammatory and immune responses as well as tumorigenesis by regulating PANoptosis (14, 37). PANoptosis is involved in various cancers; however, its effect on PC pathogenesis remains unclear.

Our results revealed that PCA could distinguish between pancreatic TT and NT based on PANRG expression, thereby indicating that PANoptosis could be involved in PC pathogenesis. Subsequently, we performed consensus clustering analysis to explore the PANoptosis-related molecular subtype, and the results showed that all patients with PC could be divided into two subtypes: PANcluster A and PANcluster B. Our results demonstrated a significant difference in the prognosis and pathways/functions enriched in patients in both subtypes. The prognosis of patients in PANcluster A was significantly worse. Moreover, the RIG I-like receptors (RLRs), cytosolic DNA, NOD-like receptor, and TLR signaling pathways, leukocyte transendothelial migration, apoptosis, the cell cycle, etc., were enriched in patients in PANcluster A. The RLRs signaling pathway functions as intracellular pattern recognition receptors to detect viral or bacterial infections and triggers host innate immune responses, thereby indicating significant involvement in anti-tumor immune responses (39). The RLRs signaling pathway induces interferon production, which causes cell death or regulates the apoptosis pathways (40–46). Furthermore, activating the RLR signaling pathway *via* lentivirus or synthetic ligands in tumor cells could induce cancer cell death with or without IFN involvement. TLRs are pattern recognition receptors expressed by immune cells and cancer cells. Moreover, TLR expression was associated with cancer progression (47–50). Activating specific TLRs, such as TLR 2, 4, and 9, increases the release of pro-inflammatory factors that promotes cancer cell metastasis and aid tumor cells in escaping immune surveillance. Interestingly, activating TLR 3, 5, and 7 induces cancer cell death, thereby attenuating cancer progression (46). Moreover, activating TLRs could increase vascular permeability by directly or indirectly recruiting leukocytes, which triggers NK cells and cytotoxic T cells to eliminate tumor cells, thereby causing tumor regression (51, 52).

TME is a complex structure composed of stroma as well as cancer, endothelial, and immune cells. Further, dynamic interactions and crosstalk occur between these cells in TME. TIME, including innate and adaptive immune cells, extracellular immune factors, and molecules on the cell surface, are critically involved in tumorigenesis (53–55). Studies have that macrophages, DCs, neutrophils, myeloid suppressor cells, NK cells, innate lymphocytes, and cytokines in TME could interfere with immune function, inhibit anti-tumor immune response mediated by T cells, stimulate angiogenesis, and promote the proliferative, invasive and metastatic ability of cancer cells (56–59). Our results showed a significant increase in macrophages, resting and activated DCs, as well as activated mast cell infiltration in patients in PANcluster A. A study has shown an increase in the levels of mast cells in TME during the early stage of tumor development, and the infiltration of mast cells correlates with PC progression (60). Studies have shown that the accumulation of pro-inflammatory immune cells, such as neutrophils and mast cells in the TME could significantly increase the migratory ability and angiogenesis in PC (61–63). Soucek et al. showed that inhibiting mast cell infiltration in pancreatic islet β-cell tumors could induce hypoxia and tumor as well as endothelial cell death (64). Compared to PANcluster A, a significantly high infiltration of naive B cells, monocytes, and CD8+ T cells was observed in patients in PANcluster B. In TME, B cells exert an anti-tumor effect by secreting tumor-specific antibodies in response to antigen presentation or acting as antigen-presenting cells (APC) to stimulate T cells. Together, this leads to T cell-mediated cytotoxicity, which restricts the growth of tumor cells. In addition, B cells can directly kill tumor cells and produce granzyme B when the B cell receptor recognizes tumor cell antigens (65). CD8^+^ T cells exert an immune response and directly eliminate damaged cells; thus, CD8^+^ T cells regulate the immune response induced by immunotherapy (66). Immune cells, such as monocytes, mediate crosstalk between innate and adaptive immune responses. Further, monocytes can affect TME *via* various mechanisms, induce immune tolerance, the proliferation of cancer cells, angiogenesis, and trigger anti-tumor responses by activating APC (67).

Apart from surgery, patients with PC, especially advanced PC, are treated using adjuvant drug therapy. However, the responses of different patients to various drugs are different. Therefore, determining the patient’s sensitivity to drugs is crucial for designing personalized treatment and improving the efficacy and response to drug therapy. Therefore, we evaluated the differences in the sensitivity of patients in the PANoptosis-related molecular subtypes to drugs. The results demonstrated higher sensitivity of patients in PANcluster A to erlotinib, selumetinib, and trametinib. These drugs are low molecular weight tyrosine kinase inhibitors that reversibly inhibit the tyrosine kinase domain of intracellular EGFR by competitively binding to ATP (68, 69). Clinical trials in European, American, and Asian populations showed that compared to conventional cytotoxic chemotherapy, the response rates and progression-free survival of patients with EGFR-mutated PC treated with erlotinib were significantly better (70–74). Patients in PANcluster B showed higher sensitivity to irinotecan, oxaliplatin, and sorafenib. Oxaliplatin, a platinum-based intercalating agent, and irinotecan, a topoisomerase inhibitor, are widely used for treating patients with cancer, including PC. Multiple studies have demonstrated that the FOLFIRINOX regimen, including the combination of irinotecan, oxaliplatin, 5-fluorouracil, and leucovorin, was more effective in treating the patient with both localized and advanced PC compared to gemcitabine monotherapy (75, 76). Therefore, designing personalized treatment strategies based on the PANoptosis-related molecular subtypes has the potential to improve the patient’s prognosis.

To enhance the ability to predict the prognosis and characteristics of patients with PC, we established and validated a PANoptosis-related prognostic model consisting of two genes: *CXCL10* and *ITGB6*. *CXCL10* is a chemokine and a low molecular weight protein secreted by cells. *CXCL10* binds to specific G protein-coupled receptors containing 23 transmembrane structural domains to induce cellular chemotaxis (77). A study has shown that chemokines could regulate angiogenesis and promote or inhibit the growth, invasion, and metastasis of cancer cells by affecting cancer cells or indirectly by recruiting immune cells to TME (78). Interestingly, *CXCL10* plays a role in immune dysfunction, chronic inflammation, and tumorigenesis and regulates the changes in TME; hence, *CXCL10* could be a new immunotherapeutic target (79). *CXCL10* binds to the CXCR3 receptor to exert its effect. Moreover, *CXCL10* exerts a dual effect on cancer progression based on the type of CXCR3 receptor (80). CXCR3-A, a major subtype of the CXCR3 receptor, benefits cell proliferation. An increase in the expression of CXCR3-A and its ligand *CXCL10* induces calcium influx in cells and enhances the invasive and migratory potentials of cancer cells *via* the p38/MAPK, ERK1/2, and JNK signaling pathways (79, 81). However, CXCR3-B exerts opposite effects. The binding of *CXCL10* to CXCR3-B inhibits the proliferative and migratory ability of tumor cells and immune response (82, 83). *CXCL10* is secreted by stromal cells in TME, and the survival of patients expressing high *CXCL10* levels was poor (84). In PC, *CXCL10* and *CCL21* increase pain due to cancer by promoting the migration of PC cells to sensory neurons (85, 86). *ITGB6* is a member of the integrin superfamily and a transmembrane heterodimer glycoprotein. In healthy adults, the epithelial cells do not express *ITGB6*, or *ITGB6* expression is low. However, high *ITGB6* expression regulates various cellular processes, ECM, and cytoskeletal interactions, including fibrosis, cell proliferation, carcinogenesis, and immune response (87–91). In gastric cancer tissues, a correlation was observed between *ITGB6* and matrix metalloproteinase 9. Further, *ITGB6* could be a downstream effector molecule of vascular endothelial growth factor, thereby enhancing gastric cancer aggressiveness; hence, *ITGB6* could be a novel biomarker for gastric cancer (92–96). Our results revealed an increase in *ITGB6* expression in pancreatic TT. Further, the prognosis of patients expressing high *ITGB6* levels was poor. Thus, *ITGB6* could be a new target for diagnosing and treating patients with PC.

To the best of our knowledge, our study is the first to use bioinformatics and experimental tools to determine the involvement of PANRGs in PCs. We have determined the association between PANoptosis and the prognosis, biological behavior, TIME, and drug sensitivity in patients with PC by constructing a PANoptosis-related molecular subtype and prognostic model. However, we retrospectively analyzed data obtained using publicly available databases. Given the rigor of our study and the novelty of the prognostic model, our results should be validated in prospective multicenter studies. Moreover, additional experimental studies are needed to uncover the underlying mechanism of the correlation between PANRGs and PC development.

## Conclusion

In conclusion, we identified the PANoptosis-related molecular subtypes and constructed a prognostic model, which was correlated with the prognosis, clinicopathological features, biological processes, TIME, and drug sensitivity in patients with PC. Our results would aid in exploring the underlying mechanism of PANoptosis in PC pathogenesis and designing a personalized therapeutic strategy for treating patients with PC.

## Data availability statement

The datasets presented in this study can be found in online repositories. The names of the repository/repositories and accession number(s) can be found in the article/**Supplementary Material**.

## Ethics statement

The studies involving human participants were reviewed and approved by Institutional Review Board of Dalian Medical University. The patients/participants provided their written informed consent to participate in this study.

## Author contributions

BZ, BH, and DS conceptualized and designed the study. BZ and BH collected the data from the database. BZ, SL, and XZ conducted data analysis. BZ, BH, and XZ wrote the article. BZ, BH, XC, and HS conducted experiments, and XC, HS, and DS revised the manuscript. All authors contributed to the article and approved the submitted version.

## Supplementary material

Supplementary Figure 1 The CDF curve when clustering number k = 2–9.

Supplementary Figure 2 Relative change of the area under the CDF curve.

Supplementary Figure 3 Univariate Cox regression analysis showed that 64 PANDEGs were associated with the prognosis of patients with PC.
