# Supplementary figures and images for: PANoptosis-related molecular subtype and prognostic model associated with the immune microenvironment and individualized therapy in pancreatic cancer
Source: Front Oncol. 2023 Jul 14;13:1217654. doi: 10.3389/fonc.2023.1217654 (PMC10382139; doi:10.3389/fonc.2023.1217654)

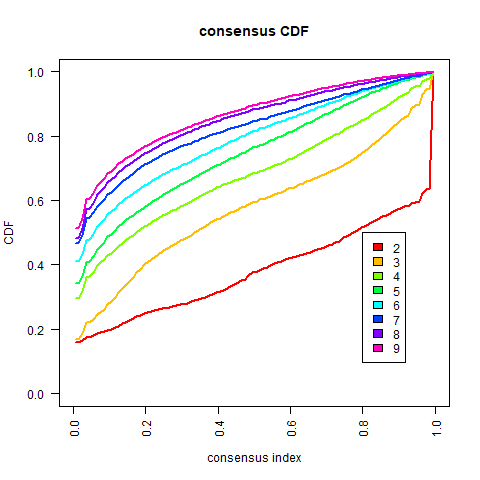

Supplement: Supplementary Figure 1 — The CDF curve when clustering number k = 2–9. [file Image_1.jpeg]

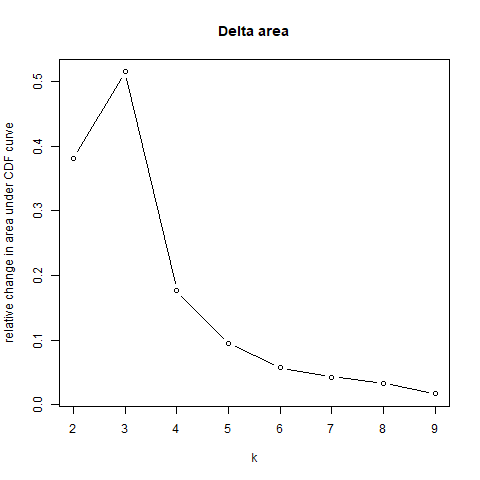

Supplement: Supplementary Figure 2 — Relative change of the area under the CDF curve. [file Image_2.jpeg]

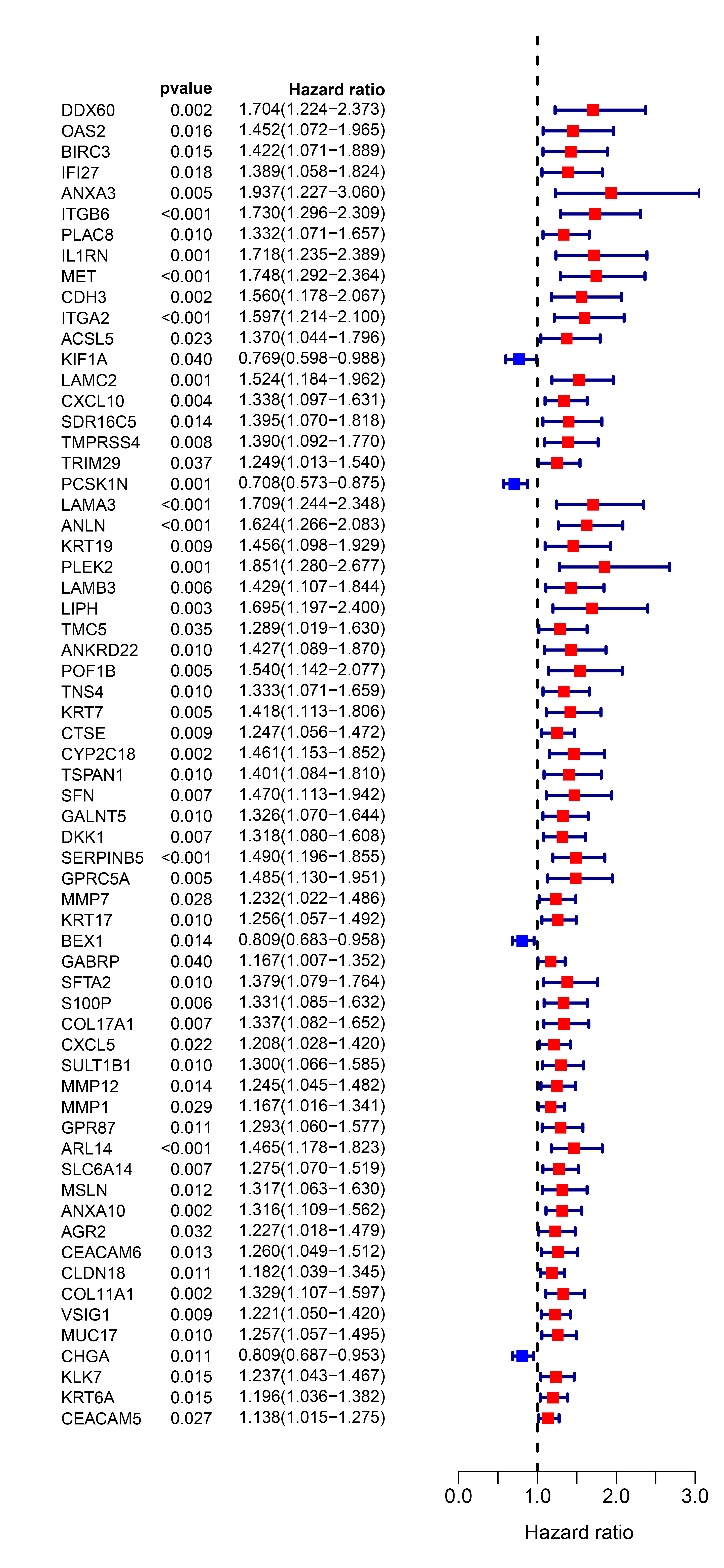

Supplement: Supplementary Figure 3 — Univariate Cox regression analysis showed that 64 PANDEGs were associated with the prognosis of patients with PC. [file Image_3.jpeg]
